# Ionizing Radiation-Induced Immune and Inflammatory Reactions in the Brain

**DOI:** 10.3389/fimmu.2017.00517

**Published:** 2017-05-05

**Authors:** Katalin Lumniczky, Tünde Szatmári, Géza Sáfrány

**Affiliations:** ^1^Division of Radiation Medicine, National Public Health Centre, National Research Directorate for Radiobiology and Radiohygiene, Budapest, Hungary

**Keywords:** ionizing radiation, cognitive effects, neuroinflammation, immune reactions, low-dose radiation

## Abstract

Radiation-induced late brain injury consisting of vascular abnormalities, demyelination, white matter necrosis, and cognitive impairment has been described in patients subjected to cranial radiotherapy for brain tumors. Accumulating evidence suggests that various degrees of cognitive deficit can develop after much lower doses of ionizing radiation, as well. The pathophysiological mechanisms underlying these alterations are not elucidated so far. A permanent deficit in neurogenesis, chronic microvascular alterations, and blood–brain barrier dysfunctionality are considered among the main causative factors. Chronic neuroinflammation and altered immune reactions in the brain, which are inherent complications of brain irradiation, have also been directly implicated in the development of cognitive decline after radiation. This review aims to give a comprehensive overview on radiation-induced immune alterations and inflammatory reactions in the brain and summarizes how these processes can influence cognitive performance. The available data on the risk of low-dose radiation exposure in the development of cognitive impairment and the underlying mechanisms are also discussed.

## Introduction

**Figure 1:**
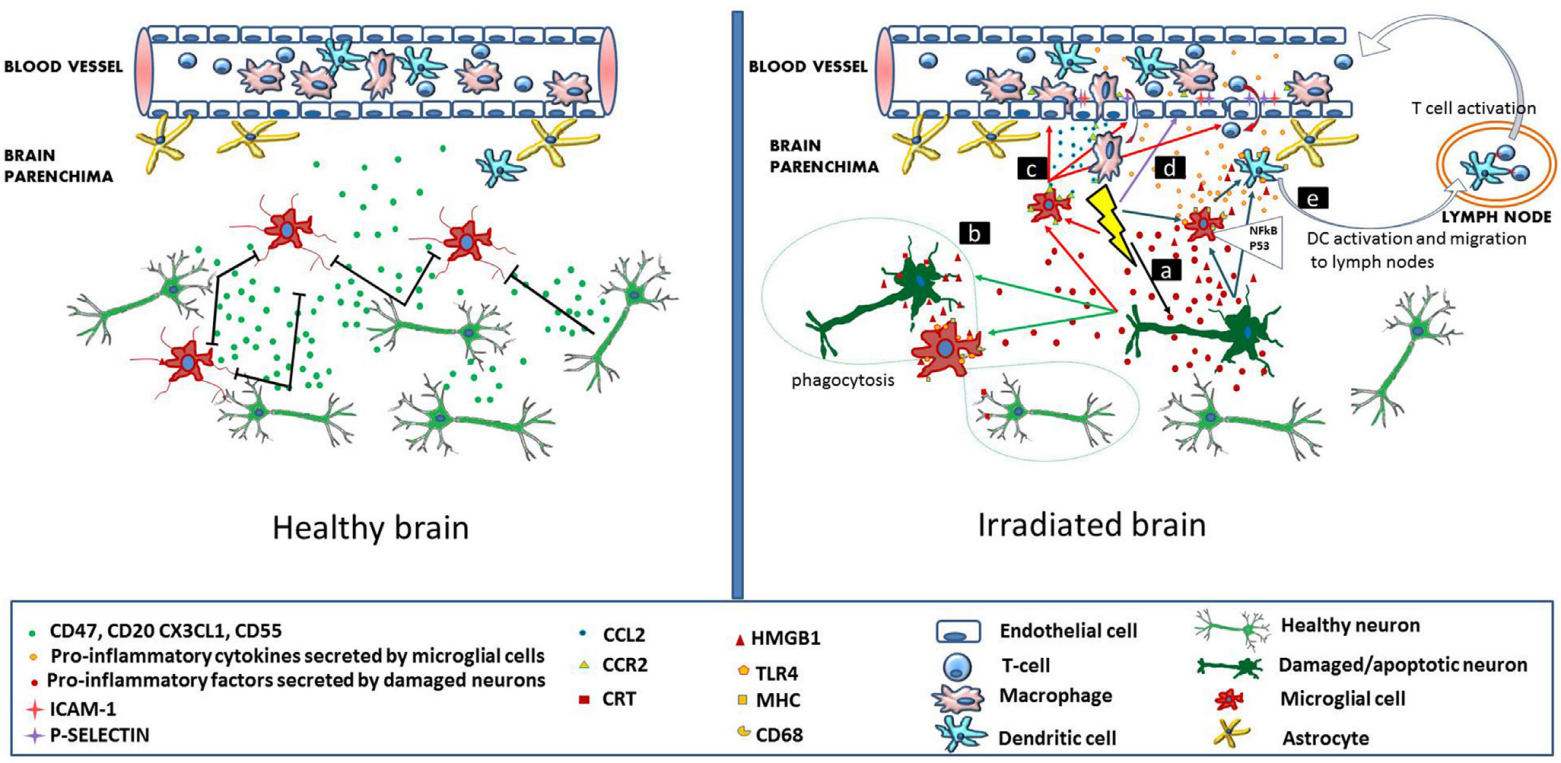
**Immune signaling in the healthy and irradiated brain**. In the healthy brain (left panel), intact neurons express and secrete molecules (CD47, CD55, CD20, and CX3CL1), which maintain adjacent microglial cells in a resting state. Brain microvascular endothelial cells, also in a resting state allow the continuous flow of blood lymphocytes and myeloid cells. In the irradiated brain (right panel), radiation-induced direct cellular damage affects neurons and microglia. Neuronal damage leads to the secretion of pro-inflammatory cytokines by the neurons, which activate microglia (mechanism a). In microglia, radiation-induced DNA damage through the NFκB pathway leads to microglia activation (MHC, CD68 upregulation) and secretion of pro-inflammatory cytokines (mechanism a). Damaged neurons secrete high-mobility group protein 1 (HMGB1) in the extracellular environment, which is a ligand for TLR4 on the activated microglia. Damaged neurons also express calreticulin on their surface, which is sensed by activated microglia and induces phagocytosis of both damaged and healthy neurons (mechanism b). Irradiation increases the secretion of CCL2 by activated microglia and also upregulates CCR2 expression. CCL2 signaling is a chemoattractant for CCR2-expressing peripheral macrophages, which penetrate the blood–brain barrier (mechanism c). Radiation induces upregulation of adhesion markers [intercellular adhesion molecule 1 (ICAM-1), P-selectin] on brain microvascular endothelial cells. Peripheral lymphocytes and monocytes adhere to activated endothelial cells and transmigrate through the microvessel wall (mechanism d). Pro-inflammatory signals and HMGB1 emitted by damaged neurons and activated microglia activate brain-residing dendritic cells, which migrate to regional lymph nodes and induce immune activation (mechanism e).

Cellular and molecular mechanisms leading to radiation-induced brain injury are far from being understood. Currently, the concomitant involvement of multiple processes is thought to contribute to the development of several pathologies. Such processes are damage at the level of microvessels leading to blood–brain barrier (BBB) leakage, increased neuronal stem, and progenitor cell death as a consequence of direct cytotoxic effect of radiation, perturbations in the energy production due to mitochondrial damage, as well as direct (activation of microglia cells) and consequential (increased infiltration of immune and inflammatory cells through the damaged BBB) inflammatory and immune reactions. Although these processes are often discussed separately for didactic purposes, they are tightly interrelated where inflammation constitutes a major link. This review will focus on the role of inflammatory and immune reactions in the development of radiation-induced cognitive deficits (Figure 1).

## The Immune Status of the Healthy Brain

Physiologically inflammation and subsequent immune reactions are protective mechanisms of the body by which foreign pathogens and damaged cells are eliminated and homeostasis is restored. During an inflammatory reaction, cellular and tissue damage of various extents takes place, which in the case of a tissue with a good regenerative capacity does not normally lead to functional deficit. Brain, however, is an organ with a very poor regenerative capacity. Thus, in order to minimize inflammation-induced neuronal damage, the interaction between the central nervous system (CNS) and the immune system is in several aspects different from other organs. This leads to a privileged immune status of the brain maintained by certain structural and functional features: (1) the BBB and the blood–cerebrospinal fluid barrier (BCSFB) are well-structured barrier systems that tightly control the free penetration of immune cells into the brain parenchyma. (2) Antigen presentation within the brain and at the regional lymph nodes is restricted due to (i) the absence of constitutive expression of major histocompatibility I molecules on neurons of the adult brain (1); (ii) the low number of professional antigen-presenting cells (APCs)—mainly dendritic cells (DCs)—and resident T cells in the brain parenchyma (2); and (iii) the lack of lymphatic vessels in the brain parenchyma, which would drain CNS-related antigens and APCs directly to the regional lymph nodes (3).

Microglial cells resident in the brain parenchyma are the main cellular components involved in the innate immune response. These cells possess professional antigen-presenting like characteristics, and as such show multiple similarities with DCs and macrophages. By expressing MHC molecules, microglial cells are capable of antigen presentation. Physiologically these are self-antigens and induce tolerance. Microglial cells also express danger-associated molecular pattern (DAMP) receptors able to sense various danger signals from their environment, such as infectious agents, molecular toxins, and cellular damage and by which they can trigger innate immune processes (4, 5). Microglia are inactive under normal circumstances which is partly due to a panel of anti-inflammatory factors (such as CD200, CX3CL1, CD47, and CD55) secreted by healthy neurons. However, they become activated by various chemokines, cytokines, and purine metabolites released by damaged neurons (6). The interaction between the microglia and neurons highlights the pivotal role of microglial cells in the immune surveillance of normal brain. However, microglial cells are relatively weak antigen presenters, and at present it is thought that contrary to DCs they cannot migrate to peripheral lymphoid organs to induce specific immune response (2). Thus, DCs are indispensable for a successful immune surveillance.

Conventional DCs are also present in certain well-defined brain regions in varying numbers. These are the juxtavascular spaces of the brain parenchyma, brain regions that physiologically lack an intact BBB, brain parenchyma in close contact with the cerebrospinal fluid (CSF) (along the ventricles) and the choroid plexus (CP) (7). A possible way for brain-residing DCs to become activated is through danger signals released by neuronal or other cellular damage in the brain parenchyma. Extracellular vesicles (exosomes and microvesicles) secreted by various cellular components of the brain parenchyma can also transmit inflammatory and activating signals toward DCs and other professional APCs situated around the microvessels and in the CSF (8). These signals are carried most probably by the interstitial fluid circulating in the direction of brain microvessels (9).

The presence of lymphocytes in the healthy brain is very scarce and mostly consists of CD4^+^ T cells and rare CD8^+^ T cells. A significant fraction of these lymphocytes are CD4^+^ memory cells. They can be found in the CSF, in the meningeal spaces, and in the stroma of the CP (the space between the blood vessel endothelium and the epithelial layer of the CP) where they continuously screen APCs presenting their cognate antigens (3). Entry of T cells at the level of the epithelial layer of the CP is facilitated by the expression of adhesion molecules such as intercellular adhesion molecule 1 (ICAM-1) and vascular cell adhesion molecule 1 (VCAM-1) by the CP epithelial cells (10). Baruch et al. have demonstrated significant enrichment of CNS-specific T cell receptor clones within the CD4^+^ T cells residing in the CP stroma (11). This means that CP-residing CD4^+^ T cells are continuously challenged by CNS-related antigens. This phenomenon was termed as “neuroprotective autoimmunity” (12), and at present it is widely accepted that it has a fundamental role in brain regenerative processes and thus it is indispensable for the maintenance of a healthy brain homeostasis (13, 14). A tightly regulated cytokine milieu within the CP is responsible for keeping the equilibrium between protective and pathological autoimmunity. This cytokine milieu mainly consists of IFN-γ and low levels of IL-4 (11, 15, 16), indicating the presence of both Th1 and Th2 lymphocytes in the CP. Wolf et al. showed in an organotypic *in vitro* model using hippocampal slice cultures that both Th1 and Th2 lymphocytes could prevent neuronal damage but the neuroprotective effect of Th2 cells was superior (17). Accumulating evidence indicates that the lack of this protective autoimmunity leads to impaired hippocampal neurogenesis, cognitive deficit, and the development of neurodegenerative disorders (18, 19).

## The Concept of Neuroinflammation: Neurological Pathologies with an Inflammatory Component

Neuroinflammation can be caused by exogenous (various infectious agents capable of invading the brain) and by endogenous factors (cellular damage within the brain parenchyma). Ionizing radiation, by causing various extent of cellular damage in the brain, is an important endogenous factor in inducing neuroinflammation.

The first step in mounting an acute inflammatory reaction within the brain consists of microglia and astrocyte activation, which sense neuronal damage in their environment. It has been already mentioned that neurons express soluble factors that inhibit microglia activation (Figure 1). It is most likely that a CNS insult leading to neuronal damage and/or death reduces/eliminates this suppression. Microglial cells remove cellular debris through phagocytosis, upregulate their MHC molecules (enhancing antigen presentation), and together with the astrocytes secrete a panel of pro-inflammatory cytokines (among others: TNFα, IL-1β, and IL-6), chemokines (CX3CL1 or fractalkine, CCL3 or macrophage inhibitory factor 1), reactive oxygen, and nitrogen species (ROS and RNS), which activate the brain-resident APCs (20, 21). It should be mentioned that while there is a certain level of immune cell trafficking through the BCSFB under physiological conditions (as detailed above), the BBB is physiologically impermeable to immune cells. However, these same cytokines can lead to endothelial cell activation and a subsequent increase in BBB permeability, as well as an increased penetrability of the BCSFB (22).

Once activated, brain-residing DCs migrate to the regional lymph nodes by the lymphatic drainage of the CSF (23), where they interact with T cells and immune activation takes place. Activated T cells reach the CNS and penetrate into the brain parenchyma *via* the altered barrier systems. A certain level of lymphocyte infiltration within the brain parenchyma during the acute phase of a neuroinflammation is needed for a quick resolution of the inflammatory process and for rapid neuroregeneration. The immunological profile of the immune cells penetrating the brain parenchyma is an immune suppressive one, consisting of Th2 lymphocytes, regulatory T cells, and M2 macrophages, which produce IL-10 and TGFβ (24, 25). Their main role is microglia suppression.

A persistent activation of the microglia and astrocytes is the hallmark of a chronic neuroinflammation. This is believed to develop when the rate of leukocyte infiltration during an acute inflammatory process is not sufficiently abundant to halt the process. A prolonged activation of microglia leads to a vicious circle, where secretion of pro-inflammatory cytokines and other neurotoxic agents (ROS and RNS) leads to further neuronal damage and cell death, which maintains microglial cells in their activated status. Interestingly, systemic immune suppressive strategies for resolving neuroinflammation are often counterproductive because they inhibit CD4^+^ T cell activation, which is needed for these cells to enter brain parenchyma and resolve the inflammatory process as described above (14).

Chronic neuroinflammation has been shown in the aging brain. In many aspects, this process is driven by and resembles systemic immune senescence, which is also an accompanying process of aging. During aging, the equilibrium between systemic immune-stimulating and -suppressive mechanisms is shifted toward immune suppression with an increase in the systemic ratio of regulatory T cells and CD4^+^ cells with a Th2 phenotype and elevated T cell anergy (26). These systemic changes impede an efficient resolution of neuroinflammation. A similar process resembling systemic immune senescence takes place within the CP as well. The level of IFNγ production by the CP epithelial cells and residing immune cells decreases and is replaced by IFNI production, while IL-4 secretion increases. This drives an increase in the production of the CCL11 chemokine by the epithelial cells, which in turn negatively regulates neurogenesis and induces cognitive decline (27).

An inflammatory component has long been known in the pathophysiology of several neurodegenerative diseases (28) and increasing evidence suggests that inflammation is involved in the pathophysiology of neurovascular and certain psychiatric disorders, as well (29, 30). The inflammation-related mechanistic link between these different diseases has been recently reviewed by several research groups. They show that DAMP-associated activation of inflammasomes in various cell types within the brain (mainly microglia, astrocytes, neurons, and endothelial cells) constitute a common mechanism in the development of different types of neurological and psychiatric disorders (31, 32). In Alzheimer’s disease, for example, where extracellular deposition of the β-amyloid (Aβ) peptide, forming the typical neuritic plaque is a major hallmark of the disease, it seems that Aβ represents a DAMP for microglial cells and causes their continuous activation through their toll-like receptors 2, 4, 9 (TLR-2, -4, -9) (33). Increased ROS levels, as activators of inflammasomes have been recognized in certain cerebrovascular diseases, whereas animal data and limited human studies indicate TLR-triggered activation of the inflammasomes in depression, bipolar disorders, and other psychological diseases (34–36). It has not been yet clarified whether inflammatory reactions are the cause or the consequence of these diseases. However, several research groups showed that in the case of Alzheimer’s disease inflammatory reactions were present already at an early stage of the disease before the appearance of the neurofibrillary pathology, suggesting a causative effect for inflammation (37, 38).

## Radiation-Induced Late Brain Injuries

Therapeutic and diagnostic medical interventions represent the main source of radiation exposure for the brain. Radiotherapy constitutes a first-line treatment option for various primary or metastatic brain tumors, as well as head and neck cancers, where a high dose (on average 50–60 Gy) is delivered in multiple fractions of approximately 1.8–2 Gy either to the whole brain or to restricted brain regions. Despite the fact that treatment schedules are planned in a way to avoid or minimize toxic side effects in healthy tissues, they still occur in a certain number of sensitive patients.

Classically, radiation-induced brain damage can be divided into acute, early delayed, and late injury based on the time of onset and includes both morphologic and functional deficits (39). Acute damage manifests itself as headache and drowsiness within hours to days after radiotherapy and is caused by brain edema. It is a fully reversible condition, and it appears rarely with modern radiation techniques. Early delayed injury is characterized by somnolence, short-term memory loss, and attention deficits and morphologically by transient demyelination. These are transient symptoms, which resolve in approximately 3 weeks without leading to long-lasting cognitive disturbances. The so-called radiation somnolence syndrome has been described mainly in children receiving whole brain radiotherapy for brain tumor treatment or prophylactic irradiation for acute lymphoid leukemia. Although it has been attributed to a transient demyelination process, recent evidence supports the inflammatory nature of this condition, where various pro-inflammatory cytokines (most notably IL-1β) play an important role. This is further supported by the fact that steroid administration can improve the symptoms (40, 41).

Radiation-induced late brain injuries develop more than 6 months after irradiation and are mostly irreversible changes. Morphological damage consists of vascular abnormalities, demyelination, gliosis, and in extreme cases white matter necrosis. Functionally, it is associated with two main alterations: endocrinopathy and cognitive impairment. Endocrinopathy develops mainly after higher radiation doses delivered to the hypothalamic–pituitary axis. Its most frequent manifestations are hypothyroidism (due to direct radiation damage on the thyroid or to decreased production of the thyroid-stimulating hormone or TSH as a consequence of radiation damage to the hypophysis), growth retardation (due to growth hormone deficiency), and gonadal dysfunction (due to gonadotropin deficiency). Several comprehensive reviews have been published in this topic (42–45). Since it is out of the scope of this review to detail radiation-induced endocrine dysfunctions, we recommend all interested readers to consult these. Cognitive deficit manifests itself in various degrees of memory impairment, learning difficulties, declined flexibility in thinking and IQ performance, and, in extreme cases, full dementia. Radiation-induced cognitive impairment is the most debilitating late sequel of brain irradiation, and it has a great impact on the quality of life of the individuals. Importantly, it often develops even in the absence of detectable morphological abnormalities (46).

Certain patient cohorts can be used to study radiation-induced cognitive impairment. Long-term survivors of glioma constitute an important group; however, in this case, a number of confounding factors (such as short follow-up period due to limited survival rates, neuropsychological symptoms attributable to the malignant disease, the impact of chemotherapy) make the correct evaluation of radiotherapy effects on the cognitive performance more difficult. Within this group, low-grade glioma patients’ follow-up is of particular interest due to their much better prognosis in terms of overall survival. Most studies agree that radiotherapy poses a significant risk of late cognitive impairment in adult patients with low-grade gliomas (47, 48), but conclusions are contradictory whether focal radiotherapy with fractional doses less than 2 Gy is associated with an increased risk of cognitive deficit (49, 50). In long-term survivors of childhood brain tumors, on the other hand, there is an agreement in the literature that the most important risk factor for impaired intellectual outcome is radiotherapy, especially in children irradiated before the age of 15 (51–53), indicating the higher vulnerability of pediatric patients to brain irradiation.

The brain can be exposed to substantial doses of irradiation during the radiotherapy of various head and neck tumors as well. However, in these cases, radiation exposure is restricted to certain brain regions only. Various trials demonstrated an increased risk of cognitive impairment in these patients (54). Meyers et al. studied the cognitive performance of patients who received paranasal sinus irradiation, where the mean delivered dose was 60 Gy in fractions of 1.8–2 Gy. They found memory impairment in 80%, learning difficulties in more than 50%, difficulty with visual–motor speed, frontal lobe executive functions, and fine motor coordination in more than 30% of the patients. Cognitive performance could be correlated with total dose delivered to the brain but not with the volume of the irradiated brain or chemotherapy treatment (55). Severe cognitive deficit was reported also in children treated with radiotherapy for head and neck rhabdomyosarcoma with symptoms manifesting within 10 years after radiotherapy (56).

Lung cancer patients receiving prophylactic brain irradiation to reduce the rate of brain metastasis are also at risk for developing late cognitive alterations (57, 58). Children with acute lymphoid leukemia constitute another important study group who, for prophylactic reasons, received cranial irradiation. In a study conducted at the Children’s Hospital in Philadelphia in the early 80s, an average total dose of 24 Gy cranial irradiation, combined with intrathecal methotrexate were applied to these children. The authors demonstrated significant reduction in the overall IQ score for the majority of children, younger patients being more affected. Notably, even in those patients who did not have any IQ decline, learning deficit was still present. However, cognitive deficits were absent in children treated with intrathecal chemotherapy only (59), indicating that chemotherapy *per se* was not a toxic agent for cognitive outcome. Waber et al. reported slightly different findings in a study conducted 15 years later at the Dana Farber Cancer Institute. In this study, cranial irradiation could not be directly linked with cognitive damage, most probably because the applied average total dose was much lower (18 Gy) (60).

It is very important to note that all of these cohorts were treated with conventional X-ray or gamma ray techniques. While major technical improvements were done to reduce irradiation of healthy tissues (such as the development of different intensity-modulated radiotherapy techniques), due to the energy deposition characteristics of these radiation types it is impossible to completely spare non-tumorous tissues. Proton radiation therapy has emerged as a novel therapeutic modality that is beginning to be largely applied for the treatment of various brain tumors. Protons are charged particles, which deposit their energy over a narrow range, and have little lateral scatters in the tissues. Due to these properties, the proton beam focuses on the tumor and doses delivered to surrounding normal tissues are much lower than in the case of X-ray-based techniques. While proton beam therapy (PBT) is a relatively new technology, and there are no large patient cohorts yet which allow a thorough evaluation of the developing side effects in the brain, the already available data indicate its suitability to reduce late toxicities. This is especially important in children whose brain is very sensitive to irradiation (as discussed above). An essential dose reduction by using PBT compared to conformal radiotherapy was shown particularly in contralaterally located critical neuronal structures (61). Different clinical studies measured superior quality of life, physical, and IQ scores in children with brain tumors receiving PBT compared to those treated with X-rays (62–65). However, all of these studies agree that additional long-term data and larger cohorts are needed to correctly evaluate the impact of PBT on neurocognitive performance and to determine whether PBT is associated with a clinically relevant cognitive sparing compared to X-ray protocols.

All the abovementioned clinical studies demonstrate that cognitive impairment is a relatively frequent consequence of high-dose therapeutic brain irradiation. While the severity of the damage is influenced by multiple factors, the most important ones are the young age at irradiation and the irradiated brain region. The exquisite sensitivity of the hippocampus to irradiation, where the neuronal stem cells are located has been shown by numerous animal experiments (66–69) and clinical studies (67, 70, 71), and it is evidenced also by the fact that the most common neurological alterations are hippocampal-related memory deficits. On the other hand, as stated by Greene-Schloesser et al. in a recent review (39), hippocampal sparing radiotherapy might not be sufficient to avoid cognitive impairment since brain regions other than hippocampus are also involved in cognitive processes. Furthermore, neuronal stem cell death is only one component in the mechanism of radiation-induced brain injury.

## Inflammation-Mediated Mechanisms in Radiation-Induced Brain Injury

### Radiation-Induced Activation of the Microglia

It is well established that ionizing radiation induces inflammatory reactions in the brain mainly *via* microglia and endothelial cell activation (72) (Figure 1). A possible mechanism on how microglia are activated is by IR-induced double-strand breaks, which trigger the NFκB pathway-mediated production of inflammatory proteins (73). Microglial cells in their activated state secrete a panel of pro-inflammatory cytokines, which inhibit neurogenesis in the hippocampus by disrupting neurogenic signaling pathways. It was shown that neuroinflammation induced a long-term disruption of hippocampal network activity and had a significant impact on the recruitment of adult-born neurons into hippocampal networks encoding spatial information. Increased levels of cyclooxygenase-2, IL-1β, IL-6, IL-18, TNFα, and interferon-gamma-inducible protein-10, as well as several chemokines such as monocyte chemoattractant protein (MCP-1/CCL2) and macrophage inflammatory protein 2 (MIP-2/CXCL2) were measured in microglial cells after radiation doses higher than 7 Gy both *in vitro* and *in vivo* (72, 74–77). Microglia activation was detected even months after irradiation indicating the persistence of the neuroinflammatory process (78). Selective inhibition of microglia-mediated neuroinflammation was able to ameliorate radiation-induced late cognitive impairment (79). Schindler et al. investigated radiation-induced neuronal loss and microglial activation in young, adult, and aged rats. They found that in younger animals 10 Gy whole brain irradiation induced a more pronounced and persistent reduction in the number of immature neurons than in aged rats. On the other hand, microglial activation was more prevalent in older animals, where 10 weeks after irradiation the proportion of activated/resting microglial cells was 60%, compared to a rate of 20% found in young animals (80). Furthermore, irradiation induced an RNA expression profile resembling to the transcriptome of the aging microglia (81). These findings are very important in our opinion since they highlight that the mechanisms responsible for radiation-induced cognitive impairment might be different in young and aged individuals. While at young age radiation-induced direct alteration in neurogenesis is the major factor, at older ages the preponderant mechanism for the development of radiation-induced cognitive deficit is neuroinflammation, which in turn impacts neurogenesis. These findings are in concordance with other reports indicating that radiation induces a premature aging process in the brain and accelerates and/or aggravates the onset of chronic degenerative disorders characteristic for elderly (82, 83).

Chemokine receptors, due to their central role in attracting immune cells to the site of inflammation, are considered as key components in mediating neuroinflammation. A panel of chemokine receptors and their ligands such as CCL7, CCL8, CCL12, CXCL4, CCR1, and CCR2 were shown to be upregulated as a result of brain irradiation (84). Among these, CCR2 has a prominent role in enhancing macrophage infiltration at the sites of injury in the brain (85) and in modulating several neurodegenerative disorders (86). It was postulated that irradiation influenced neurogenesis and cognitive functions by altering CCR2 signaling pathways in the brain. Recently, Belarbi et al. proved the direct involvement of CCR2 expression in the development of radiation-induced cognitive alterations. Using CCR2 knockout mice, they showed that CCR2 deficiency prevented cranial irradiation-induced neuronal damage and cognitive impairment (84). The protective effect of CCR2 deficiency against radiation-induced neuronal damage was identified after low-dose irradiation, as well (doses below 2 Gy) (87).

Since the phenotype of activated microglia is difficult to discern from brain-infiltrating activated macrophages (88), it is possible that the main inflammatory cells within the brain parenchyma are originating from blood-derived macrophages penetrating into the brain parenchyma, which becomes permissive for them in an inflamed state. Several lines of evidence support this hypothesis. Burrell et al. demonstrated that bone marrow-derived cells were recruited specifically to the site of cranial irradiation in a dose-dependent manner and differentiated predominantly into inflammatory cells and microglia (89). Mildner et al. conducted a very elegant experiment in which they proved the role of cranial irradiation in the engraftment of blood-derived macrophages into the brain parenchyma. They identified a specific monocyte subpopulation (Ly-6C^hi^Gr-1 + CCR2 + CX3CR1^lo^ cells), as the precursor of adult murine microglia in the peripheral blood and showed that microglia engraftment during postnatal life was enhanced by various degenerative brain disorders. However, these monocytes were preferentially recruited to the brain and differentiated into microglia only if the brain was “preconditioned” by irradiation. The authors explained this enhanced cell engraftment primarily by a radiation-induced production of CCL2 in the brain, which attracted blood-derived CCR2-expressing monocytes and by an inactivation of the repository signals and to a lesser extent by a radiation-induced damage in BBB integrity, although they admitted that subtle BBB alterations might have been present (90). Similar findings were reported by Lampron et al., who induced myeloablation either by chemotherapy or by total body irradiation and followed the repopulation of the hematopoietic niche, as well as the entry of bone marrow-derived cells into the brain. While repopulation was equally efficient after both chemo- and radioablation, brain penetration of bone marrow cells was only observed after irradiation (91). Morganti et al. showed that a single dose of cranial irradiation with 10 Gy induced a significant decrease in brain-residing microglia, while significantly increasing the penetration of blood-derived CCR2^+^ macrophages. They also proved that penetrating macrophages adopted a microglia-like phenotype. Similar to Mildner et al., they also did not detect BBB damage, which could be responsible for the increased penetration of monocytes, but demonstrated a radiation-induced increase in the secretion of a panel of chemoattractant molecules implicated in the recruitment, adhesion, and migration of monocytes (92). On the other hand, it seems that repopulation of brain parenchyma with peripheral microglia progenitors does not necessarily happen under physiological conditions, since these bone marrow-residing progenitors do not mobilize spontaneously to the peripheral blood and can only reach the CNS if artificially delivered into the circulation (93).

The way a cell is dying greatly impacts the immune and inflammatory response of the host. The characteristics of an immunogenic cell death have been initially described for cancer cells (94). One of the most important features of an immunogenic cell death is that dying cells expose so-called “eat-me” signals sensed by nearby tissue-residing phagocytes (95) and the physiologically present phagocytic barrier is lost. CD47 is considered a typical phagocytic barrier or “don’t eat me” signal, which in the context of cellular apoptosis is frequently lost and this phenomenon is paralleled with the cell surface exposure of the endoplasmatic reticulum-associated calreticulin (CRT) (96). Cell surface bound CRT is the most important “eat-me” signal for surrounding phagocytes. It seems that “eat-me” and “don’t eat me” signaling molecules are present in neurons as well, indicating that interactions between neurons and activated microglia are in multiple aspects similar to those seen outside the brain (97). Although the presence of cell surface CRT is usually characteristic for dying cells, it has been shown that neurons constitutively express it (98). Resting microglia do not react with CRT-expressing neurons. However, as shown by Fricker et al., microglia activation *via* ligands binding to their TLR4 receptor has led to the phagocytosis of CRT-expressing both viable and apoptotic neurons, significantly contributing to the amplification of a neurodegenerative condition (98). Irradiation can impact this process in multiple ways. Radiation induces apoptosis among neuronal stem and progenitor cells (99). Whether IR-induced apoptosis is *de facto* accompanied by increased cell surface CRT levels on neurons has not been reported yet, but it has been shown in carcinoma cells (100), and this phenomenon was directly linked with the induction of an immunogenic type of apoptotic cell death (101). Experiments related to CD47 changes in apoptotic cells after ionizing radiation are also lacking. However, it was shown that UV-induced apoptosis induced CD47 redistribution on the cell surface associated with a significant reduction in the binding efficiency of CD47 to its natural ligand on phagocytes. This resulted in facilitating the clearance of apoptotic cells by phagocytes (102).

We have previously discussed that IR can directly activate microglial cells. It is very probable that IR can contribute to microglia activation *via* their TLR4 receptor as well. The prototypic TLR4 ligand is lipopolysaccharide (LPS), which is an endotoxin released by bacterial cells during an infection. On the other hand, the endogenous LPS-like molecule high-mobility group protein 1 (HMGB1) is a danger signal (or alarmin), which is released in the extracellular medium under cellular stress. It was shown that HMGB1 by binding to the TLR4 receptor could promote microglia activation under stress conditions associated with neuronal damage such as traumatic brain injury, ischemic injury, and methamphetamine treatment (103–105). Studies investigating the direct effect of IR on HMGB1 release and TLR4 activation in the brain are not available yet. However, given the fact that IR is a strong cellular stressor, it is plausible to hypothesize that it induces similar stress-related pathways than other stressors.

### Radiation Effects on Brain Endothelial Cells, BBB Integrity, and Immune Cell Infiltration in the Brain

Blood–brain barrier is a major route for the systemic supply of immune and inflammatory cells during neuroinflammation. There are not too many *in vivo* studies referring to the impact of acute cranial irradiation on BBB integrity. The previously mentioned studies reported no significant BBB damage after high-dose irradiation (around 10 Gy), though they did not exclude the possibility of minor BBB alterations (90, 92). On the other hand, other studies detected significant alterations in BBB damage with or without alterations in endothelial tight junctions after high-dose irradiation, albeit this damage was transient, and its severity varied in the different brain regions (106–108). *In vitro* models also demonstrated that alterations in BBB integrity were detected after much lower doses (4 Gy). These alterations were relatively long lasting and were accompanied by increased permeability for both low- and high-molecular weight proteins. Morphologically, a rarefaction of the endothelial layer was seen, which could lead to the opening of the endothelial tight junctions, despite the fact that no gross alterations were observed in the immunolabelling of a panel of tight junction proteins (ZO-1, claudin-5, and occludin) (109).

Endothelial cells are among the most radiosensitive cellular structures in the brain. Direct IR induces endothelial cell death by various mechanisms. Several *in vitro* and *in vivo* studies demonstrated endothelial cell apoptosis as an early event after irradiation. However, it was induced only by high doses of irradiation and was accompanied by strong inhibition of endothelial cell proliferation capacity (110, 111). The rate of apoptotic endothelial cells was estimated to be around 15% within 24 h after irradiation with high doses (112, 113). Li et al. demonstrated a direct link between radiation-induced endothelial cell apoptosis and acute increase in BBB permeability (110).

Recently, it has been shown that senescence is another major cell death mechanism developing at a later time point in the surviving endothelial cells. Irradiation doses in the range of 2–8 Gy led to increased DNA damage and a reduced repair efficiency in rat primary cerebrovascular endothelial cells, which were accompanied by increased yields of endothelial cells showing premature senescence and acquiring a senescence-associated secretory profile. Endothelial senescence could be a consequence of pro-inflammatory cytokines secreted by activated glial cells and astrocytes such as TNFα or IL-6 (114, 115). These senescent cells acquired certain phenotypical features resembling activated endothelial cells. Senescent endothelial cells significantly contributed to the onset and progression of neuroinflammation by secreting a panel of pro-inflammatory molecules (IL-1α, IL-6, and MCP1), upregulating adhesion molecules on their surface, and increasing their ROS production (116, 117).

Changes in the activation status of microvascular brain endothelial cells can facilitate immune cell transmigration even in the absence of an overt BBB damage. Several studies reported that high doses of IR could directly activate brain microvascular endothelial cells by increasing ICAM-1, VCAM-1, and P-selectin expression (118–120). ICAM-1 induction on brain endothelial cells is a rapid but persistent process, appearing as soon as 4 h after irradiation and being detectable even 6 months later (119, 121). Since ICAM-1 expression has a major role in facilitating leukocyte trafficking into the brain parenchyma, its persistent presence contributes to the slow resolution of the neuroinflammatory process. Another important molecule regulating monocyte and leukocyte transmigration through the BBB is CD47 expressed on endothelial cells. CD47 plays an active role in immune cell diapedesis by interacting with the signal-regulatory protein alpha on monocytes, activating signaling pathways that induce cytoskeleton remodeling and cadherin redistribution. CD47 activation was shown to occur after ischemic neurovascular injury, and its overexpression on brain endothelial cells significantly enhanced monocyte transmigration and contributed to BBB injury and edema (122–124). It remains to be determined whether radiation injury to the brain induces similar CD47 changes.

Moravan et al. performed a systematic longitudinal analysis of brain-infiltrating immune cells after irradiation. According to this study, neutrophil penetration in the irradiated brain was a transient effect, which could be detected only in the first 12 h after irradiation. CD3^+^ T cells penetrated the brain as early as day 7 after irradiation and persisted even 12 months later. DC penetration was also seen, and similar to T cells, it was a rather late process persisting up to 6 months after irradiation. Several of the penetrating DCs acquired an activated phenotype and often colocalized with T cells suggesting a possible interaction between the two cell types. Penetration of myeloid cells in the brain was dose dependent within the range of 5–35 Gy radiation dose and was dependent on CCR2 signaling (121, 125).

## Low-Dose Radiation Effects on the Brain

The vast majority of radiation exposures delivered to the brain in the population are for diagnostic purposes, where absorbed doses are in the low-dose range (below 100 mGy). Recent epidemiological data pose serious concerns regarding long-term health consequences of these low doses. It was shown by several epidemiological studies that cranial CT exposure increased the risk of brain tumors in children (126–128). Similar conclusions were drawn after interventional radiology exposures to the brain (127) as well as in hemangioma cohorts subjected to head irradiation for hemangioma treatment (129). A recent report indicated a twofold increased risk of brain cancer mortality among technologists who performed fluoroscopically guided interventional procedures (130). These observations raise the possibility that low-dose radiation might cause cognitive alterations as well. We found one report in the literature about the risk of late cognitive deficit in humans subjected to low-dose cranial irradiation. A population-based cohort study was performed in Sweden involving 3,030 boys who were treated with IR for cutaneous hemangioma before the age of 18. The study could not show any difference regarding logical, spatial, and technical test scores between IR-treated subjects and controls, but verbal test scores displayed a significant trend for decreasing scores with increasing doses to the hippocampus. The authors also concluded that hippocampal dose was a better predictor of late cognitive side effects than doses delivered to other brain regions (131). While human epidemiological data are almost absent, several animal experiments indicate cognitive damage as a potential long-term risk of low-dose cranial irradiation. Altered adult spontaneous behavior and impaired habituation capacity was found in mice exposed to low doses (500 mGy) total-body irradiation at a very young age (postnatal day 3 and 10) but not later, indicating an exquisite sensitivity of the young brain to IR. The same group showed significantly higher alterations in the behavior of these mice if they were coexposed to IR and nicotine (132, 133). Gene expression studies performed in the brain or various brain structures repeatedly report mRNA expression profiles characteristic for low-dose exposure. Low-dose exposures (100 mGy) induced genes that were not affected by high doses (2 Gy), and low-dose genes were associated with unique pathways and functions similar to those seen in the aging brain and in the brain tissue from patients with Alzheimer’s disease (134). Yin et al. also showed qualitatively different gene expression profiles after 0.1 and 2 Gy, where low-dose-regulated genes were involved in protective and reparative functions such as stress response, cell cycle control, and DNA repair as well as in neural signaling activity (135). Dose-dependent changes in gene expression profiles were seen in human neuronal progenitor cells, where very low-dose chronic irradiation (31 mGy/72 h) induced alterations in inflammatory pathways related to interferon signaling, while higher doses induced different signaling pathways (136). It was reported that low-dose chronic irradiation stimulated leptin production in mice (137, 138). Leptin is a member of the cytokine superfamily, resembling IL-6 also known as the “saturation hormone” produced mainly by adipocytes. It acts on receptors in the hypothalamus to inhibit hunger and thus has major role in maintaining a metabolic balance. It has important effects on the immune system as well, by shifting the Th1/Th2 balance in favor of Th1 cells, by regulating monocyte–macrophage activation, by inducing T cell proliferation, and by suppressing apoptosis (139). Since leptin levels were directly correlated with cognitive performance and higher leptin levels could even ameliorate cognitive deterioration seen in Alzheimer’s disease (140–142), low-dose radiation-induced increase in circulating leptins might be a favorable parameter in the risk of radiation-induced cognitive alterations.

Very interesting data start to emerge regarding the impact of low-dose or low-dose rate irradiation on endothelial cell integrity. A premature senescence was observed in human umbilical vein endothelial cells exposed to low-dose rate irradiation delivered by 2.4 or 4.1 mGy/h dose rates. Transcriptomic and proteomic studies revealed the activation of signaling pathways related to cell–cell communication, adhesion, and inflammation in these cells with a special involvement of the insulin-like growth factor-binding protein 5 in this process (143, 144). Endothelial damage in the brain was reflected in a rarefaction of capillary density after low-dose (0.1 Gy) whole brain irradiation (66). These data indicate that doses well below those considered damaging for various brain structures lead to microvascular disturbances and endothelial dysfunction promoting the onset of a neuroinflammatory process.

Exposition of astronauts to cosmic rays during deep space flights represents another source of low-dose irradiation to the brain. Cosmic rays are mainly composed of high-atomic number and energy charged particles (high-energy protons and fully ionized atomic nuclei). These are densely ionizing radiations, which differ from main terrestrial radiation types (X and γ-rays) in terms of biological damage. The density of ionizing events deposited in tissues by charged particles produces a track of biological damage (mostly complex DNA double-strand breaks), which is very difficult to be repaired through the cellular repair processes. Exposure to heavy ion irradiation as low as 0.5 Gy was supposed to induce impaired neurogenesis with a very poor or no recovery (145). A long-lasting functional damage induced by low-dose heavy particles was shown in the hippocampus, leading to cell type-specific alterations in both the excitatory and inhibitory synaptic microcircuits (146). Significant dose-dependent and long-lasting reductions in dendritic complexity, spine density, and morphology (147) as well as altered neurogenesis (148) were observed in hippocampal neurons after low-dose total-body proton irradiation. At molecular level, long-term changes in DNA methylation patterns (149), distinctive miRNA signatures (150) were described in the brain following proton irradiation. Similar to γ-rays, heavy ion exposure also increased circulating leptin levels (151). It was reported by Baluchamy et al. that high-energy protons induced a dose-dependent increase in reactive oxygen species and lipid peroxidation as well as a reduction in antioxidant levels in the brain, mainly in the neural stem cells, followed by apoptotic cell death (152–155).

Very few studies investigated the effect of low doses of proton and heavy ion irradiation on inflammatory and immune parameters in the brain. Vlkolinsky et al. showed that LPS treatment of mice in the absence of (56)Fe-particle irradiation induced a reduction in the hippocampal long-term potentiation capacity, while this inhibition was abolished and a reversal effect was registered after irradiation of the brain with (56)Fe ions. This phenomenon persisted for months, indicating that heavy ion irradiation stably altered hippocampal reactivity to immunological stressors (156). Regarding the direct effect of protons or heavy ions on brain inflammation existing reports are contradictory. Raber et al. demonstrated microglia activation in the hippocampus of mice exposed to low-dose proton, heavy ion, or combined irradiation, which correlated well with deterioration in novel object recognition, suggesting a role for neuroinflammation in the development of cognitive impairment (157). On the other hand, Sweet et al., investigating low-dose effects of high-energy proton particles on inflammatory reactions in the hippocampus, could not detect significant astrocyte and microglia activation indicating lack of neuroinflammation. They also found significantly reduced ICAM-1 levels selectively in the hippocampus, pointing to a lack of endothelial activation and/or to a capillary rarefaction and endothelial cell loss (148).

## Conclusion

In this review, we presented data proving a direct link between ionizing radiation-induced neuroinflammation and the development of late neurodegenerative disorders and cognitive deficit. It has been shown that the most common radiation-related alterations after brain irradiation are various forms of cognitive deficit. Some of the most representative epidemiological cohorts presenting an elevated risk for late cognitive sequela have been reviewed highlighting the increased sensitivity of the developing brain (and thus children) for radiation damage. The second part of the review focused on the description of the mechanisms on how IR can induce inflammatory reactions and can perturb brain immune homeostasis. IR-induced neuroinflammation develops as a result of a complex signaling between various cellular components residing in the brain (neurons, microglia, astrocytes, and endothelial cells) as well as the peripheral immune system. These data clearly prove that immune reactions in the brain are in many aspects similar to systemic immune reactions. Finally, we have discussed the long-term risk of low-dose radiation on the brain and presented the already available epidemiological and experimental data supporting this increased risk. These findings showed that molecular and cellular mechanisms within the low-dose range are often different from those elicited by high-dose irradiation. The relevance of these data is huge, since this means that even doses in the range used for diagnostic purposes might have long-lasting consequences and might contribute to the development of radiation-induced late cognitive impairment.

Although much progress has been made in the field, the mechanisms that govern IR-induced inflammatory and immune reactions in the brain, their relationship with IR-related functional deficit and consequently the optimal therapeutic countermeasures are far from being elucidated. While formerly research work focused almost exclusively on therapeutic radiation doses, new and accumulating data regarding the risk of low-dose radiation highlight the importance of studies within this dose range as well.

## Author Contributions

KL designed the review. KL, TS, and GS wrote the review.

## Conflict of Interest Statement

The authors declare that the research was conducted in the absence of any commercial or financial relationships that could be construed as a potential conflict of interest.
